# Bacterial-Induced Blood Pressure Reduction: Mechanisms for the Treatment of Hypertension *via* the Gut

**DOI:** 10.3389/fcvm.2021.721393

**Published:** 2021-08-13

**Authors:** Tyler Alexander Cookson

**Affiliations:** Department of Physiology, University of Alberta, Edmonton, AB, Canada

**Keywords:** hypertension, gut microbiome, butyrate, probiotics, inflammation, cardiovascular disease, dysbiosis

## Abstract

Hypertension is a major risk factor for the development of cardiovascular disease. As more research into the gut microbiome emerges, we are finding increasing evidence to support that these microbes may have significant positive and negative effects on blood pressure and associated disorders. The bacterial-derived metabolites that are produced in the gut are capable of widespread effects to several tissue types and organs in the body. It is clear that the extensive metabolic function that is lost with gut dysbiosis is unlikely to be replenished with a single metabolite or bacterial strain. Instead, combinations of bacteria and concomitant therapies will provide a more well-rounded solution to manage hypertension. The bioactive molecules that are recognized in this review will inform on ideal characteristics of candidate bacteria and provide direction for future research on the gut microbiome in hypertension.

## Introduction

Hypertension is a significant risk factor for development of cardiovascular disease—the leading cause of death worldwide (1). It is believed that the gut microbiome may be involved in the development and the perpetuation of the hypertensive phenotype. The use of live bacteria has the potential to confer a wider array of functionality compared to supplementation of a few metabolites, such as short-chain fatty acids (SCFAs). Additionally, due to the widespread changes observed in the abundance of gut microbes during hypertension (2–6), it is unlikely that supplementation of a single bacterial strain will completely normalize blood pressure in a hypertensive patient. Using a combination of species and strains as probiotics has the potential to temporarily restore metabolic function that is lost in hypertensive microbial dysbiosis. The gut microbiome is complex and a group of strains may provide additional functionality through cooperation, such as cross-feeding, and reintroduce a multi-faceted solution in which many components work in concert to treat hypertension in a way that could not be achieved by supplementing a single metabolite or bacterial strain.

Notably, as germ-free animals exhibit marked reductions in blood pressure (7), there are likely species of gut bacteria that promote the development of hypertension. Therefore, identifying properties of bacteria that can facilitate reductions in blood pressure is important for future research. This review will discuss the potential involvement of various gut metabolites and processes in the development and perpetuation of hypertension and propose a comprehensive mechanism for a bacterial-induced blood pressure reduction to serve as a guide for properties of bacteria to investigate further.

## Dietary Bioactive Molecules Synthesized and Metabolized by Gut Microbes With Effects on Blood Pressure

### Butyrate, Acetate, Lactate, and Isovalerate

Along with acetate and propionate, butyrate is one of the main SCFAs produced by gut microbes in the gut. Currently, butyrate is being studied primarily for its anti-inflammatory properties (8, 9) and intestinal barrier activity through tight junction expression (10, 11). However, newer research questions the efficacy of butyrate in human tissue (12). There is early evidence to support that butyrate promotes reductions in blood pressure, but additional research is required to determine the validity of these results in human models.

In hypertensive patients, researchers have observed depleted circulating butyrate (13). When butyrate is infused intracolonically in rats, there was a more profound hypotensive effect compared to when butyrate was infused intravenously (14). Additionally, the intracolonic butyrate reduced heart rate (HR) in these rats, while the intravenous butyrate did not, suggesting that butyrate can stimulate a central hypotensive effect *via* pathways in the gut. Interestingly, butyrate activates Gpr41/43 and OR51E1 receptors on enterochromaffin cells (ECCs) to release serotonin (5-HT) (15–17), which binds to 5-HT_3_ receptors on vagal afferents (18). As activation of vagal afferents can stimulate glutamate release at the nucleus tractus solitarius (NTS) of the brain (19, 20), it is possible that the glutamate in the NTS can elicit a central hypotensive effect (19). While this pathway is only a hypothesis, it may help to explain the hypotensive effects observed by intracolonic butyrate that is not seen with intravenous infusion and should be investigated further. Importantly, chronic stimulation of ECCs by butyrate increases 5-HT synthesis (15), but acute exposure does not (21), indicating a need for consistent supplementation either directly or *via* production by probiotics. This pathway may explain the profound hypotensive effect and the reduction in HR that is observed with intracolonic butyrate, but not intravenous butyrate. Although 5-HT may have a role in the central hypotensive effects mediated by the gut-brain axis as described above, high circulating levels of 5-HT can also constrict blood vessels and contribute to hypertension (22, 23).

It should be noted that intravenous butyrate does still have a hypotensive effect. Additional research has shown that butyrate can inhibit sympathetic tone in peripheral blood vessels, including the mesenteric arteries and the gracilis muscle arteries, *via* the Gpr41/43 receptors (14) and reduce norepinephrine (NE) production by destabilizing tyrosine hydroxylase mRNA stability (24) to attenuate vasoconstriction. Butyrate also dilates blood vessels independent of the Gpr41/43 receptors (25), potentially through the OR51E1 receptors present in vascular smooth muscle and renal vasculature (26, 27). Moreover, butyrate suppresses angiotensin II (AngII)-induced hypertension by inhibiting renal prorenin receptors and the intrarenal renin-angiotensin system (RAS) (28). In the brain, intracerebroventricular injection of butyrate can also centrally reduce blood pressure directly by altering neuronal activity in cardio-regulatory regions of the brain (29).

Some researchers suggest that in a subset of hypertensive patients, butyrate uptake is impaired in the proximal colon, such as that which is observed in spontaneously hypertensive rats (SHRs) (29, 30). This theoretically results in an increase in intracolonic butyrate, and therefore an increase in 5-HT release from ECCs. Under normal conditions, this would be expected to reduce sympathetic output centrally, however, it is possible that the hypotensive effect is dampened due to reduced sensing mechanisms such as lower proportions of appropriate receptors in the brain. For example, SHRs have reduced “SCFA-sensing receptors” in the brain that would normally aid in producing a hypotensive effect (29). Interestingly, fecal transplant from a normotensive rat into an SHR not only normalized blood pressure, but also increased Gpr41 and Gpr43 receptors in the brain (31). Therefore, supplementation of certain bacteria or combinations of bacteria may not only have mechanisms to reduce blood pressure metabolically, but also re-sensitize the body to hypotensive signals by altering receptor expression. Bacteria from the *Faecalibacterium, Clostridium, Eubacterium*, and *Roseburia* genera are major butyrate producers, but many species of bacteria are capable of butyrate production (32, 33).

The primary use for acetate and lactate in this proposed pathway is for the production of butyrate. For example, species of *Clostridium* can utilize acetate and lactate to produce butyrate (34). However, acetate is known to activate a parasympathetic response (35), although the exact means by which this is accomplished has yet to be elucidated. Acetate is known to bind to Gpr41 and Gpr43 (36), and may act through the ECCs in a similar fashion to butyrate. Importantly, acetate can reduce blood pressure (37), but whether this is *via* its own actions, through butyrate production, or a combination is also not known. In addition to promoting butyrate production, acetate can downregulate the metabolism of butyrate in the kidney to maintain its availability and prolong its actions (37). Much less is known about the branched short-chain fatty acid (BSCFA) isovalerate. From what is known, isovalerate can activate OR51E1 on ECCs, which is similar to butyrate and may contribute to a centrally induced hypotensive response (16). Additional actions of isovalerate in the context of blood pressure needs to be explored further to determine the magnitude of its effects, if there are any at all.

### Propionate

In addition to butyrate, propionate is another major SCFA produced in the gut and is therefore a metabolite of high interest regarding its effects in the body, especially in disease states. Recently, a study evaluating the effects of propionate on hypertensive cardiac damage and atherosclerosis found that propionate decreased blood pressure, reduced systemic inflammation, improved vascular dysfunction, and attenuated atherosclerosis in mice *via* reductions in T_H_17 cells (38). This study suggests that the effects of propionate on blood pressure and cardiovascular damage are largely T-cell dependent, but have yet to be described in great detail. However, it is known that propionate can have a direct effect on blood pressure that also happens to be dose-dependent. Lower concentrations of propionate will activate Gpr41 and decrease blood pressure, while higher concentrations will activate Olfr78 (with OR51E2 being the human homolog) to increase blood pressure *via* renin signaling in the renal juxtaglomerular apparatus (39, 40). In addition to Gpr41, propionate can also activate Gpr43 similarly to butyrate (36) and therefore might also stimulate 5-HT release from ECCs. While this has not been investigated explicitly, a Gpr43 knockout model with supplementation of propionate will clarify the extent to which propionate affects blood pressure through the Gpr43 receptor.

Concerning the production of propionate by gut bacteria, certain strains of *Enterococci* can utilize lipids, such as cholesterol, to produce propionate which can reduce the development of atherosclerosis (41). Importantly, the growth of *Enterococci* is inhibited by propionate (42), potentially as a feedback mechanism to limit propionate production by the *Enterococci* that may prevent an excess of propionate from increasing blood pressure. It should be noted that butyrate, succinate, and lactate can be converted to propionate (43), so increased levels of propionate may occur through butyrate, succinate, or lactate conversion rather than direct synthesis.

### Gamma-Aminobutyric Acid

Similar to butyrate, circulating Gamma-Aminobutyric Acid (GABA) is also reduced in hypertensive patients (30). Luminal GABA in the gut binds to GABA_B_ receptors on ECCs, stimulates 5-HT release, attenuates the sympathetic response, and has a hypotensive effect (44). In addition, it should be noted that germ-free mice, which demonstrate reduced blood pressure (7), also have depleted GABA in the stool and the blood (45) suggesting that changes in circulating and gut luminal GABA is not wholly responsible for the observed changes in blood pressure, if at all. Activation of GABA_B_ receptors on vagal afferents has been described to inhibit afferent signaling (46). It would be expected from this that higher concentrations of intra-luminal GABA would increase transport of GABA across the intestinal barrier and blunt vagal afferent activity; however, there is a strong inverse relationship between intraluminal GABA and blood pressure (44) that suggests the hypotensive effects of GABA outweigh the inhibitory activity on the vagal afferents. The effects of GABAergic signaling in the gut are still controversial and should be explored further; however, additional beneficial roles such as reducing inflammation (47) have been described for GABA in the gut.

### Lipopolysaccharide and Gut Inflammation

Intestinal inflammation is suggested to play a large in part in the development of hypertension and cardiovascular disorders as endothelial dysfunction is observed in arteries located far from the inflamed intestines, such as in inflammatory bowel diseases (48). Moreover, intestinal inflammation promotes microbial dysbiosis and the breakdown of commensal bacteria (49) that are required for normal vascular physiology (50). High levels of Lipopolysaccharide (LPS), a major component of the outer membrane of Gram-negative bacteria, in the intestinal lumen degrades tight junction proteins and compromises gut barrier integrity (51, 52) which can potentially be rescued by butyrate (53). A consequence of reduced gut barrier function is “leaky gut,” in which the permeability between the gut lumen and the circulation is not as closely controlled. Bacterial translocation into the circulation can cause a sterile systemic inflammation as bacteria are degraded by host immune cells and release LPS (54–56). A murine model showed that injection of LPS into the periphery or directly into the brain induced neuroinflammation either by crossing the blood-brain barrier or by stimulating afferent nerves at circumventricular organs to alter blood-brain barrier permeability (57). Though still being investigated, neuroinflammation contributes to central sympathetic hyperactivity (58, 59) to promote increases in blood pressure, including neuroinflammation induced by LPS (60).

### Histamine

Histamine has strong hypotensive effects (61). By increasing the permeability of capillaries (62), histamine increases fluid extravasation, reduces blood volume, and therefore reduces blood pressure. In addition, histamine is a strong vasodilator, which contributes to the observed hypotensive effects. Of interest, histamine can suppress inflammasome signaling to attenuate gut inflammation by LPS, limit IL-18 secretion, and reduce the expression of antimicrobial peptides that promote inflammation (63) to maintain gut barrier integrity. In the heart, histamine can impair the actions of AngII by reducing AT_1_ receptor expression in the heart *via* H_3_ receptors (64).

### Bile Acids and Trimethylamine-*N*-oxide

Although a majority (95%) of bile acids are reabsorbed and recycled, gut bacteria are able to manipulate them while they are present in the gut lumen. Certain species of bacteria can convert primary bile acids to secondary bile acids, such as deoxycholic acid and lithocholic acid, which bind to TGR5 receptors on ECCs to stimulate 5-HT synthesis and release (65, 66). Trimethylamine-*N*-oxide (TMAO) is currently a highly researched metabolite in the area of hypertension. Several bacteria can promote TMAO synthesis *via* the production of trimethylamine (TMA) from phosphatidylcholine, choline, and carnitine metabolism. Unfortunately, TMAO reduces primary bile acid synthesis in the liver (67), which results in less activation of TGR5 receptors on ECCs. TMAO also contributes to hyperlipidemia by blocking reverse cholesterol transport and increasing forward cholesterol transport (67). In the vasculature, TMAO increases endothelial inflammation and inhibits endothelial nitric oxide (NO), suppressing vasodilation (68, 69).

TMAO-rich foods are also an important component of the Mediterranean diet and while TMAO is largely being investigated for its pathogenic roles, it may also have several beneficial biological functions. In the gut, TMAO serves as an electron acceptor in oxidative phosphorylation for anaerobic bacteria to reduce the production of reactive oxygen species (70) while also inhibiting the growth of pathogenic bacteria (71) suggesting that TMAO has protective functions for both bacteria and the host. In rodent models, TMAO prolonged the hypertensive effects of AngII; however, when TMAO was infused on its own, there were no noticeable negative effects (72). In fact, oral L-carnitine, which is ultimately converted into TMAO, elicits reduced markers of vascular injury (73). Thus, as TMAO on its own exhibits several positive effects and lacks strong deleterious effects in the absence of other well-known contributors to hypertension, it is unlikely to induce hypertension alone but may cooperate with other pro-hypertensive molecules, such as AngII.

### Hyperlipidemia

Elevated serum lipids or hyperlipidemia can contribute to increased blood pressure through various means, including atherosclerosis. Atherosclerosis is the buildup of plaque in the walls of blood vessels, which narrows the lumen of the blood vessel and increases blood pressure. Serum lipids can also increase oxidative stress in the vascular endothelia, thus increasing monocyte recruitment, and reducing NO availability (74, 75). As NO is a strong vasodilator, this impairs vasodilation and can further increase blood pressure. Bacteria that promote the production of secondary bile acid synthesis may induce more cholesterol use for the *de novo* synthesis of bile, resulting in less serum lipids.

In germ-free mice, there are no differences in endothelial-dependent or independent relaxation compared to conventional mice (50). This suggests that the gut microbes in conventional animals do not alter endothelial function in the absence of a prohypertensive challenge. Additionally, a lack of microbes alone cannot explain the differences in endothelial-dependent relaxation. Instead, the presence of pathogenic bacteria or a dysbiotic gut microbiome profile with unfavorable net effects on vascular endothelial function may be able to explain these differences.

### Arachidonic Acid

Many actions for arachidonic acid (AA) have been described, but many are contradictory or often dose dependent. For example, several sex differences in the actions of AA have been distinguished. Firstly, AA exacerbates inflammation in male mice, but attenuates it in females (76). Zhuang et al. (76) attributed this observation to the fact that the AA promoted the survival of butyrate-producing bacteria and upregulated Gpr41 expression in the females. Male mice also showed reduced circulating 5-HT compared to the females in response to AA, likely due to reduced butyrate activating Gpr41 on ECCs. Evidently, sex differences in the effects of many molecules exist, including in NO-dependent vascular relaxation (50), and should researched in more detail. These findings are important and could possibly describe differences that lead to sex-dependent treatments of hypertension.

In rats, gut microbiota-derived AA reduces corticosterone, the rodent equivalent of cortisol (77). This stress hormone is heavily involved in the pathophysiology of hypertension as it can be converted into aldosterone that increases salt and water retention to increase blood volume and therefore blood pressure. Unfortunately, AA is also linked to inflammation (78). Typically, AA will stimulate an inflammatory reaction, while the *n*−3 fatty acids will counteract these effects with anti-inflammatory actions. However, appropriate levels of AA appear to be beneficial, especially in certain populations. Although there is some potential for increased inflammation with arachidonic acid, the anti-inflammatory effects of other metabolites, such as the SCFAs, should strongly oppose any inflammatory response.

### Indoles

Indoles are aromatic, heterocyclic, organic compounds that are found in the gut. As a metabolite of tryptophan, the functions of indole in health and disease are becoming more widely researched. A recent study by Huć et al. (79) investigated the peripheral and central hemodynamic mechanisms of indole. When they infused indole intravenously in male rats, there was an observed dose-dependent increase in blood pressure, but no noticeable change in heart rate. However, this same study shows that intracerebroventricular infusion of indole reduces both blood pressure and heart rate. Even more interestingly, these changes were negated by pre-treatment with 5-HT receptor blockers (79). Therefore, indoles function in both the periphery and centrally *via* serotonergic mechanisms to alter blood pressure. While peripherally indoles are prohypertensive, indoles in the brain appear to be anti-hypertensive.

### Glucagon-Like Peptide 1

SCFAs have been shown to promote Glucagon-Like Peptide 1 (GLP-1) release in the gut. For example, propionate stimulates GLP-1 secretion from L-cells *via* Gpr43 (80). Moreover, butyrate and acetate stimulate secretion of GLP-1 from enteroendocrine L-cells independent of Gpr41 and Gpr43 (81). A meta-analysis found that GLP-1 and GLP-1 receptor agonists were able slightly reduce blood pressure, but GLP-1 levels were not significantly associated with hypertension (82). Additionally, although the net effect is quite minor, GLP-1 may have a dose-dependent relationship with blood pressure in which high doses can exhibit a hypotensive effect (83).

## Metabolites Contributing to Hypertension *Via* the Gut-Kidney Axis

The kidney is the final common pathway for the development of hypertension. This statement is supported by early transplantation studies in rats. In fact, transplantation of a hypertensive kidney into a normotensive animal is sufficient to increase blood pressure, and conversely, transplantation of a normotensive kidney into a hypertensive animal can normalize blood pressure (84, 85). Additionally, germ-free studies have shown that mice lacking a gut microbiome develop much more severe kidney disease (86). As such, the gut microbiome and gut-derived metabolites that directly impact kidney function and contribute to chronic kidney disease are important in the context of blood pressure regulation.

### Oxalate

There may be a link between butyrate, oxalate, and hypertension, although this area of research could be expanded upon. As less butyrate is produced in hypertension as described above, there is weakened intestinal barrier function as butyrate induces expression of tight junctions between the gut epithelia (11). With the development of a “leakier” gut, more oxalate will be absorbed into the circulation. Once in the kidney, oxalate promotes the formation of kidney stones and can damage the kidney (87), potentially impairing the long-term control of blood pressure. Additionally, kidney stone formation is associated with higher risk of developing hypertension (88). There has been a focus on the use of *O. formigenes* in preventing kidney stones as it has demonstrated the ability to directly metabolize oxalate, utilizing acetate in the process, and producing formate (89). Formate also weakly binds to the Gpr41 and Gpr43 receptors (36).

Several species of bacteria, specifically from the *Bacteroides* and *Enterococcus* genera, express the Oxalate:formate antiporter to uptake oxalate (90). However, these species have not been found to express the appropriate enzyme, formyl-CoA:oxalate CoA-transferase, for the metabolism of oxalate (90). An untested hypothesis is that these species may uptake oxalate and, since probiotics do not colonize in the gut, together the bacteria and the oxalate may be excreted, thus preventing its uptake into the circulation.

### Uremic Toxins

Uremic toxins are metabolites and waste products that are eliminated by the kidney under normal circumstances. However, as kidney function begins to decline, the kidney is unable to excrete these toxins efficiently and appropriately, resulting in their accumulation. Several uremic toxins, namely *p*-Cresol and indoxyl sulfate, are produced by gut microbes and are uptaken into the circulation where they can exhibit detrimental renal and cardiovascular effects.

A major producer of *p*-Cresol is the pathogenic bacterium *Clostridium difficile* (91). *p*-Cresol, or *p*-cresyl sulfate after it has been conjugated by gut microbes, reduces renal function by means of oxidative stress to induce damage (92, 93). It has been proposed that the microbiota-derived *p*-Cresol or *p*-cresyl sulfate can increase arterial stiffness (94, 95) and activate the intrarenal RAS (96). Indoxyl sulfate has also been associated with activation of the intrarenal RAS (96). Thus, these two uremic toxins have the potential to increase blood pressure not only through RAS-induced renal fibrosis and impaired blood pressure regulation, but also directly through the RAS and angiotensin II. However, a study strictly assessing the effect of *p*-Cresol and indoxyl sulfate supplementation on blood pressure is lacking to validate this. Both indoxyl sulfate and *p*-cresyl sulfate also contribute to inflammation as they have been associated with increased serum pro-inflammatory cytokine expression, including IL-6 for *p*-cresyl sulfate and IL-6, TNF-α, and IFN-γ for indoxyl sulfate (92, 95). Importantly, while the microbiome is protective of kidney disease, production of uremic toxins by certain bacteria can exacerbate kidney disease and have cardiovascular consequences that contribute to undesirable effects on blood pressure.

## Additional Dietary Bioactive Molecules With Hypotensive Effects

**Figure 1:**
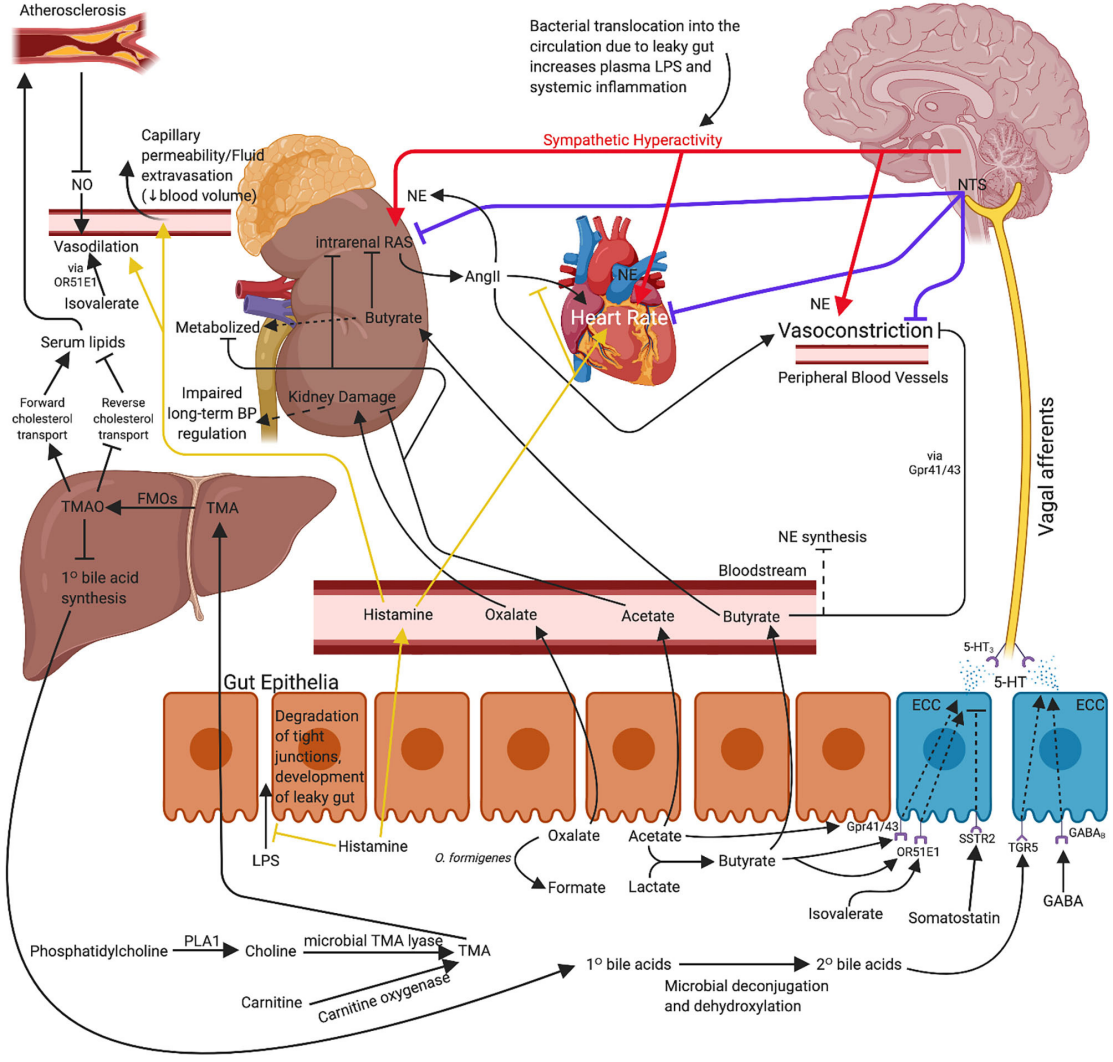
Proposed mechanism for use of probiotic bacteria and associated metabolites for the management or concomitant treatment of hypertension. The effects of propionate and arachidonic acid are not included as the exact mechanisms of action are not yet clear. Immune mechanisms are also excluded for clarity. 5-HT, serotonin; AngII, angiotensin II; BP, blood pressure; ECC, enterochromaffin cell; FMO, flavin monooxygenase; GABA, gamma-aminobutyric acid; LPS, lipopolysaccharide; NE, norepinephrine; NO, nitric oxide; NTS, nucleus tractus solitarius; *O. formigenes, Oxalobacter formigenes*; PLA1, phospholipase A1; TMA, trimethylamine; TMAO, trimethylamine-*N*-oxide. Created with BioRender.com.

In addition to the factors described above, there are numerous other molecules that may contribute to the hypotensive effects conferred by the gut microbiome. A review by Dave et al. (97) details several proteins with renin inhibitor, ACE inhibitor, and anti-oxidant activity. Notably, some of these proteins include mucins, cholecystokinin, gastrin, somatostatin, and gastric inhibitory peptide among many more. Although these other molecules are known to have hypotensive effects, research surrounding these effects and their production or metabolism by specific gut microbes is limited compared to those described in more detail above. Figure 1 proposes an illustrated mechanism for gut-derived metabolites in lowering blood pressure. Table 1 describes the general functions of the metabolites listed in this review supported by human and animal studies.

**Table 1:** A summary of gut-derived metabolites and their general functions in hypertension.

**Metabolite**	**Actions in hypertension**	**Studies**
**Short chain fatty acids**		
Acetate	• Parasympathetic activity • Butyrate production and prevents butyrate metabolism • Anti-inflammatory ° Promotes release of anti-inflammatory cytokines (e.g., IL-10) and suppresses release of pro-inflammatory cytokines (e.g., IL-12 and TNF-α)	Animal: (35, 37, 98)
Butyrate	• Anti-inflammatory properties ° Histone deacetylase activity ° Promotes release of anti-inflammatory cytokines (e.g., IL-10) and suppresses release of pro-inflammatory cytokines (e.g., IL-6, TNF-α, and IFNγ-induced iNOS) • Promotes intestinal barrier function • Capable of heart rate reduction • Intestinal 5-HT synthesis (chronically) • Limits sympathetic tone in peripheral vasculature • Reduces norepinephrine production • Capable of vasodilation • Suppresses angiotensin II production and action by inhibition of the intrarenal RAS • Intracerebroventricular injection directly reduces blood pressure centrally • Promotes vascularization	Animal: (10, 14, 24, 25, 28, 29, 31, 53, 99–101) Human tissue: (11, 12)
Lactate	• Butyrate production	
Isovalerate	• May have anti-hypertensive effects *via* the gut-brain axis	
Propionate	• Dose dependent pro- and anti-hypertensive properties ° G-protein coupled receptor and T-cell dependent mechanisms • Anti-inflammatory properties ° Reduces T_H_17 cell expression	Animal: (38–40, 101, 102)
GABA	• Anti-hypertensive *via* the gut-brain axis • Anti-inflammatory	Animal: (44–46)
Lipopolysaccharide (LPS)	• Inflammatory ° Including intestinal, neuro-, and sterile systemic inflammation ° May contribute to sympathetic hyperactivity • Promotes gut dysbiosis • Degrades intestinal barrier activity	Animal: (51, 52, 57, 60) Human: (55, 56)
Histamine	• Strong vasodilator • Reduces blood volume by fluid extravasation • Inhibits inflammatory actions of LPS • Maintains intestinal barrier activity • Counteracts the actions of angiotensin II in the heart • Increases heart rate	Animal: (62, 64)
Secondary Bile Acids (e.g., deoxycholic acid and lithocholic acid)	• Intestinal 5-HT synthesis • Anti-inflammatory ° Inhibition of IL-12 and TNF-α production	Animal: (66, 103) Human tissue: (65, 104, 105)
TMAO	• Supports hypertensive effects of an angiotensin II challenge • Reduces primary bile acid synthesis • Promotes hyperlipidemia • Increases endothelial inflammation • Suppresses vasodilation • Reduces production of reactive oxygen species • Prevents vascular injury	Animal: (67, 68, 71, 72) Human: (69, 73)
Arachidonic acid	• Pro-inflammatory in male mice; anti-inflammatory in female mice • Protective of butyrate-producing bacteria and upregulates Gpr41 receptors • Reduces corticosterone in rats	Animal: (76, 77)
Indole	• Directly, pro-hypertensive in the periphery and anti-hypertensive in the brain • Suppress T_H_17 responses ° Reduce IL-17 release • Stimulates IL-10 release from regulatory T cells	Animal: (79, 106, 107)
GLP-1	• May have a minor role in blood pressure regulation • Slightly reduces blood pressure, but not significantly associated with hypertension	Human: (82)
Oxalate	• Induces kidney stone formation and renal damage	Human: (87, 88)
**Uremic toxins**		
*p*-Cresol	• Induces renal damage by oxidative stress • Increases arterial stiffness • Activates intrarenal RAS • Pro-inflammatory ° Promotes IL-6 expression	Animal: (92, 95, 96) Human: (93, 94)
Indoxyl sulfate	• Activates intrarenal RAS • Pro-inflammatory ° Promotes IL-6, TNF-α, and IFN-γ expression	Animal: (92, 96) Human: (95)
**Polyamines**		
Spermidine	• Dietary spermidine decreases blood pressure, and vasodilates blood vessels • Reduces systemic inflammation	Animal: (108, 109) Human: (110)
Spermine	• Decreases blood pressure intravenously • Anti-inflammatory ° Suppresses IL-12, IL-18, and IFNγ expression ° Promotes IL-10 expression • Oral spermine increases blood pressure	Animal: (111–113)
Cadaverine	• Decreases blood pressure intravenously • Pro-inflammatory ° Intraperitoneal injection induces systemic inflammatory response	Animal: (111, 114)
Putrescine	• Decreases blood pressure intravenously • Anti-inflammatory ° Suppresses IL-18 and LPS-induced inflammation ° Dietary putrescine reduces pro-inflammatory infiltration into tissues • Pro-inflammatory ° Intraperitoneal injection induces systemic inflammatory response	Animal: (111, 114, 115)
Polysaccharide A	• Anti-inflammatory ° Stimulates IL-10 release ° Supresses T_H_17 responses	Animal: (116–118)

## Impact of Gut Microbiota-Derived Metabolites on Hypertension *Via* Immune Mechanisms

The lamina propria of the gut houses the largest proportion of immune cells in the body, especially T cells (119, 120). Some consider hypertension to be an autoimmune disorder, and there is a plethora of evidence to support this claim (121–123). The close relationship between the gut microbiota and differentiating immune cells may be a significant component in understanding and treating hypertension. For instance, circulating T_H_17 cells are significantly higher in hypertensive patients compared to controls (124). T_H_17 cells release the cytokine IL-17A which can have numerous effects in hypertension, such as increased salt retention that would contribute to an elevated blood volume (125). High salt can also promote activation of organum vasculosum laminae terminalis (OVLT) in the brain and stimulate yet another mechanism for central sympathetic hyperactivity (126). In addition, lesioning of the OVLT in animal models of salt-induced hypertension attenuates the increase in blood pressure (127).

### Indoles

In the context of inflammation in hypertension, indoles can inhibit T_H_17 cells and suppress the release of IL-17A, thus reducing sodium retention (128). In addition to this, indoles can bind to aryl hydrocarbon receptor precursor (AhR), half of a heterodimer nuclear receptor that modifies gene expression in T cells. Through this mechanism, indoles promote IL-10 release from regulatory T cells (Tregs) and suppress T_H_17 responses (106, 107). IL-10 is an anti-inflammatory cytokine that can improve gut barrier function by reducing gut inflammation (129). Gut microbes that express the indole-3-glycerol phosphate synthase, indole-3-glucerol phosphate lyase, and indole synthase enzymes may be able to produce indole and potentially have beneficial hypotensive effects.

### Butyrate, Acetate, and Propionate

While SCFAs have many metabolic effects, they are also able to ameliorate hypertension *via* immune-mediated mechanisms. Butyrate stabilizes hypoxia-inducible factor-1 (HIF-1), which can promote vascularization to reduce blood pressure (99, 130, 131). Additionally, HIF-1 can improve intestinal epithelial barrier integrity through lymphocytic immune responses and reduce uncontrolled permeability (99, 132). Moreover, Park et al. (100) showed that sodium butyrate, as well as sodium phenylbutyrate and sodium phenylacetate, are able to potently increase IL-10 and decrease IL-6, TNF-α, and IFNγ-induced iNOS in murine macrophages *in vitro*.

The SCFAs have been described to affect histone deacetylase (HDAC) activity. Specifically, propionate and butyrate can promote colonic Treg proliferation and differentiation by altering HDAC activity to affect gene expression (101, 102). On the other hand, acetate can ameliorate colonic inflammation through Gpr43 receptors *via* suppression of pro-inflammatory cytokines, including IL-12 and TNF-α, and promoting anti-inflammatory signals, such as IL-10 (98).

### Bile Acids

Bile acid metabolites can also contribute to immune homeostasis and limit systemic damage that is seen in inflammatory diseases. For example, secondary bile acids can act as TGR5 ligands to reduce pro-inflammatory cytokine production, including IL-12 and TNF-α, in macrophages and dendritic cells (104, 133). Furthermore, secondary bile acids can bind to the pregnane X (PXR) and farnesoid X receptors (FXR) to reduce inflammatory tissue damage (103, 105). However, it should be noted that the exact mechanism and the degree to which secondary bile acids contribute reductions in inflammation through PXR and FXR are not quite known and further research should be conducted. The anti-inflammatory actions of secondary bile acids have yet to be investigated extensively in the context of hypertension, but there is the potential that secondary bile acids could attenuate the inflammation observed in hypertension.

### Polyamines

The polyamines are not frequently discussed when evaluating disease states, however, they are quite present in the gut due to microbial fermentation and can have a slew of effects in the body, especially in immune homeostasis. The four main polyamines are spermidine, spermine, cadaverine, and putrescine.

1. Spermidine
  Dietary spermidine in human models has been shown to reduce blood pressure and arterial stiffness by directly increasing arginine bioavailability and NO, which vasodilates blood vessels (108–110). In addition to this, plasma TNF-α and systemic inflammation are reduced by spermidine intake (108).
2. Spermine
  When administered intravenously into rats, spermine reduced blood pressure in a dose-dependent manner (111). However, in rat models with oral spermine administration, there is evidence of an increase in blood pressure (112). The reason for the discrepancy in intravenous versus oral spermine on blood pressure is unclear. Research into the effects of polyamine supplementation and mechanisms of action in the context of hypertension is limited and must be explored further. Regarding inflammatory mechanisms, spermine is able to reduce inflammation by lowering production of IL-12 and interferon-γ (IFNγ), while increasing IL-10 production (113). Similarly to histamine, both spermine and putrescine can suppress the cleavage of protease caspase-1 and reduce secretion of IL-18 (63). Notably, spermine can be converted to spermidine by spermine oxidase to have additional effects.
3. Cadaverine and Putrescine
  When administered intravenously into rats, cadaverine, and putrescine both reduced blood pressure in a dose-dependent manner (111). However, in human subjects, dietary putrescine had no significant effect on blood pressure (109). Conversely, there is limited research into direct effects of cadaverine on blood pressure in human subjects. In rabbits, intraperitoneal injection of cadaverine and putrescine induced a systemic inflammatory response (114). However, dietary putrescine reduced pro-inflammatory cytokine infiltration into the intestinal walls of piglets (115). *In vitro*, putrescine ameliorates LPS-induced inflammation and pro-inflammatory cytokine release (115). Through reductions of inflammation, and specifically LPS-induced inflammation in the gut, putrescine has the potential to be protective against development of “leaky gut” and bacterial translocation. While the intravenous anti-hypertensive mechanisms of cadaverine and putrescine remain elusive, anti-inflammatory properties are evident with dietary supplementation.

### Polysaccharide A

Little is known about which bacterial species produce or express polysaccharide A (PSA), however *B. fragilis* is widely accepted to express it and can utilize PSA to interact with other microbes and host cells (134). In immune homeostasis, PSA can bind to toll-like receptor-2 (TLR2) on CD4^+^ T cells to stimulate IL-10 release from regulatory Tregs and suppress T_H_17 responses (116–118). As discussed above, hypertensive patients express more T_H_17 cells that can increase blood pressure by salt retention.

## Conclusion

Through interaction with the gut, brain, kidneys, liver, heart, vasculature, and host immunity, the gut microbiota and the metabolites which are produced, consumed, and otherwise manipulated can have widespread effects in the body. There are still many topics of research that remain to be investigated in this area, but what has become clear is that the gut microbiome is a complex and highly interactive system. For the treatment of disease states, a single “one-size fits all” metabolite or bacterial strain is unlikely to restore the extensive metabolic activity that is lost with intestinal dysbiosis in hypertension. Instead, probiotic supplementation using several bacterial strains that produce or reduce the production of appropriate metabolites to elicit a net anti-hypertensive effect is a strong potential treatment strategy for hypertension management. Additional targeted therapies, such as phage therapy, that specifically deplete or mitigate the growth of certain bacterial strains may also prove to be useful. Characterization of interactions between several bacterial strains will be important going forward to determine the best combinations of probiotic bacteria and metabolites for these new therapies.

## Author Contributions

TC performed the literature review, any relevant data collection, data analysis, and wrote the manuscript and involved in the critical revision of the manuscript and approved the final version.

## Conflict of Interest

The author declares that the research was conducted in the absence of any commercial or financial relationships that could be construed as a potential conflict of interest.

## Publisher's Note

All claims expressed in this article are solely those of the authors and do not necessarily represent those of their affiliated organizations, or those of the publisher, the editors and the reviewers. Any product that may be evaluated in this article, or claim that may be made by its manufacturer, is not guaranteed or endorsed by the publisher.
